# The diagnostic relevance of mesenteric lymph node biopsy in small intestinal lymphoma in cats

**DOI:** 10.1111/jvim.17095

**Published:** 2024-06-10

**Authors:** Laura Marconato, Valeria Martini, Barbara Banco, Silvia Benali, Veronica Crocchianti, Selina Iussich, Michele Marino, Maria Massaro, Teresa Bruna Pagano, Luca Aresu

**Affiliations:** ^1^ Department of Veterinary Medical Sciences University of Bologna BO Italy; ^2^ Department of Veterinary Medicine and Animal Sciences University of Milan Lodi Italy; ^3^ MYLAV La Vallonea Veterinary Diagnostic Laboratory Rho MI Italy; ^4^ Department of Veterinary Sciences University of Turin Grugliasco TO Italy

**Keywords:** bowel, cat, histology, immunohistochemistry

## Abstract

**Background:**

Regional lymph nodes are frequently sampled in cats with suspected intestinal lymphoma; however, their diagnostic value has not been explored.

**Objectives:**

To investigate whether histologic and immunohistochemical analysis of mesenteric lymph nodes correlates with the diagnosis of intestinal lymphoma in cats.

**Animals:**

One hundred 2 client‐owned cats diagnosed with intestinal lymphoma.

**Methods:**

Retrospective study. The inclusion criteria required a full‐thickness biopsy of the small intestine and concurrent excision of mesenteric lymph nodes. Histologic and immunophenotypic analyses were performed on intestinal biopsies and corresponding lymph nodes. Selected nodal samples diagnosed with reactive lymph nodes underwent clonality testing.

**Results:**

Transmural T‐cell lymphomas, encompassing small and large cell types, were predominant (64 cases, 62.7%), with large B‐cell lymphomas being more frequently transmural (68.8%) than mucosal (31.2%). Among all lymph nodes examined, 44 (43.1%; 95% CI: 33.9%‐52.8%) exhibited neoplastic infiltration. Among cases of small cell lymphoma, 51 out of 72 (70.8%; 95% CI: 59.4%‐80.1%) showed no nodal involvement. Clonality results correctly identified 19/30 (63.3%; 95% CI: 45.5%‐78.2%) reactive lymph nodes. Concerns were raised regarding clonal identification in the remaining cases and potential misdiagnoses based on phenotypic characteristics.

**Conclusion and Clinical Importance:**

The study underscores the potential drawbacks of relying solely on mesenteric lymph nodes for diagnosing intestinal lymphomas in cats, particularly small cell subtypes. It emphasizes the importance of full‐thickness biopsies for assessing transmural infiltration and recommends caution when utilizing mesenteric lymph nodes for histologic, immunohistochemical and clonality evaluations in mucosal lymphomas. Despite limitations, this research highlights the need for comprehensive diagnostic strategies in cats with intestinal lymphoma.

**Abbreviations**
DSH: domestic short hair
FFPE: formalin‐fixed paraffin‐embedded
HE: hematoxylin and eosin
Ig: immunoglobulin
IHC: immunohistochemistry
LN: lymph node
MLBCL: mucosal large B‐cell lymphoma
MLTCL: mucosal large T‐cell lymphoma
MSBCL: mucosal small B‐cell lymphoma
MSTCL: mucosal small T‐cell lymphoma
NS: not significant
PARR: PCR for antigen receptor rearrangement
TLBCL: transmural large B‐cell lymphoma
TLTCL: transmural large T‐cell lymphoma
TRG: T‐cell receptor gamma locus
TSBCL: transmural small B‐cell lymphoma
TSTCL: transmural small T‐cell lymphoma

## 1 INTRODUCTION

Gastrointestinal lymphoma is the most prevalent lymphoma in cats, primarily affecting the small intestine and, to a lesser extent, the large intestine and stomach.^1^ The prevailing hypothesis suggests that its origin lies within the lymphoid tissues of the digestive system, possibly originating from follicles or Peyer's patches and later disseminating to other anatomic sites.^2^

Intestinal lymphoma in cats clinically manifests with signs such as weight loss, vomiting, diarrhea, lethargy, and changes in appetite.^3^ Diagnosis typically involves a comprehensive approach, including physical examination, blood tests, diagnostic imaging, cytologic, histologic and molecular analysis of the affected intestine.^2^, ^3^, ^4^, ^5^, ^6^, ^7^, ^8^, ^9^, ^10^, ^11^, ^12^, ^13^

Recent classifications have categorized intestinal lymphoma in cats into mucosal and transmural types, based on the extent of tumor infiltration in full‐thickness biopsy samples and immunophenotype.^2^ Additional histologic features considered for diagnosis include the size of neoplastic cells, distinguishing between small cell lymphoma and intermediate‐large cell lymphoma. This differentiation is now acknowledged as the most predictive variable for prognosis, offering crucial insights into the understanding and management of intestinal lymphoma in cats.^2^

Studies on intestinal lymphoma in cats have mainly focused on the histologic and phenotypic features of mucosal or transmural lesions, with no reported data on the state of the regional lymph nodes (LNs).^2^, ^3^, ^11^ However, it is common practice to sample the regional or mesenteric LNs in cases displaying clinical signs suggestive of intestinal lymphoma. This approach is occasionally favored over endoscopic or laparotomy intestinal sampling to avoid nondiagnostic samples, to minimize the complications associated with these procedures, or both.^14^, ^15^, ^16^ Cytologic assessment of abdominal lymphadenomegaly can aid in distinguishing high‐grade lymphomas, other round‐cell neoplasms, or infectious diseases. However, because of the absence of architectural details, cytology is not effective in distinguishing small cell lymphoma from inflammatory disease processes.^1^

To date, no studies have explored the histologic status of mesenteric LNs for diagnosing and staging gastrointestinal lymphoma in cats. Therefore, we conducted a histologic and immunophenotypic analysis on a set of feline lymphomas located in the small intestine, where an excised LN was obtained during surgery. Our hypothesis suggests that histologic and immunophenotypic analysis can aid in diagnosing large cell intestinal lymphoma, but could not be as useful for small cell intestinal lymphoma.

## 2 MATERIALS AND METHODS

### 2.1 Case selection

A retrospective electronic database search was conducted using the MYLAV laboratory diagnostic service records (Milan, Italy) for reports of cats containing the terms “intestinal lymphoma” and “alimentary lymphoma” over a 6‐year period (June 1, 2017 to June 1, 2023).

Inclusion criteria were: (a) a minimum of 4 full‐thickness biopsies of the small intestine, (b) excision of the entire mesenteric LN, and (c) an immunohistochemical (IHC) panel comprising CD3 and CD20 staining on both the intestine and LN. The inclusion of a clonality test in the diagnostic workup was considered optional. Available clinical data were extracted from the accompanying biopsy submission forms.

All cats had been sampled for diagnostic purposes, with the informed consent of the owner. Thus, specific ethical committee approval to use leftover specimens for research purposes was not required (EC decision 1965/2017).

### 2.2 Histologic analysis

Formalin‐fixed small intestinal and nodal biopsies obtained from cats showing clinical suspicion of intestinal lymphoma were paraffin‐embedded (FFPE), and 4‐μm tissue sections were stained with Hematoxylin and eosin (HE).

The classification of intestinal lymphomas was determined based on the histologic variables previously published by Kiupel et al.^11^ In brief, the location was defined as mucosal (infiltrate confined to the epithelium and lamina propria with minimal extension into the submucosa) or transmural (infiltrate extending markedly into the submucosa and muscularis propria). For cases with a mucosal location, only those presenting diffuse lymphoid infiltration were included, specifically those with at least 5 plaques in each biopsy. Additionally, the neoplastic infiltrate was assessed for cell size, distinguishing between small cells (nuclear diameter <2 red blood cell diameter) and intermediate‐large cells (nuclear diameter >2 red blood cell diameter). Lymphomas with infiltrates consisting of large lymphocytes with eosinophilic cytoplasmic granules (suggestive of large granular lymphocyte lymphomas) were excluded. Epitheliotropism was also recorded.

For LN biopsies, a differentiation between reactive LN and lymphoma was primarily assessed. Reactive LN was diagnosed when the lymphoid cortex displayed enlarged follicles with prominent germinal centers, accompanied by plasma cells in the medullary cords and throughout the interfollicular parenchyma. The presence of an expanded paracortex was also considered.^17^ Neoplastic infiltration of the LN was diagnosed when a diffuse lymphoid population, either of large cells or small cells, effaced the entire architecture of the LN. However, the final diagnosis was always confirmed after the IHC characterization of the lymphoid population and its distribution within the LN.

### 2.3 Immunohistochemistry

For IHC, consecutive FFPE sections obtained both from the intestine and LN were processed as previously described.^2^ Immunohistochemistry with anti‐human CD3 and CD20 antibodies was conducted using a BenchMark ULTRA Ventana (Medical Systems, Tucson, USA). Detailed information regarding the panel of primary antibodies is provided in Supplementary Table 1. Immunohistochemical analysis adhered to the guidelines of the American Association of Veterinary Diagnosticians Subcommittee on Standardization of Immunohistochemistry.^18^ Cross‐reactivity of these antibodies has been previously confirmed. Negative controls were obtained by incubating duplicate sections of the positive control tissues with the anti‐mouse IgG1 (Code X0931, Dako, Glostrup, Denmark), which was diluted to match the same concentration of mouse IgG as the primary antibody.

For the small intestine biopsies, the diagnoses were categorized based on the combination of histologic and IHC features as follows: “mucosal small T‐cell lymphoma” (MSTCL), “mucosal large T‐cell lymphoma” (MLTCL), “mucosal small B‐cell lymphoma” (MSBCL), “mucosal large B‐cell lymphoma” (MLBCL), “transmural small T‐cell lymphoma” (TSTCL), “transmural small B‐cell lymphoma” (TSBCL), “transmural large T‐cell lymphoma” (TLTCL), or “transmural large B‐cell lymphoma” (TLBCL).

Regarding the LN biopsies, diagnoses were categorized as “reactive LN,” “diffuse small T‐cell lymphoma,” “diffuse small B‐cell lymphoma,” “diffuse large T‐cell lymphoma,” or “diffuse large B‐cell lymphoma.”

### 2.4 Clonality

Polymerase chain reaction (PCR) for antigen receptor rearrangement (PARR) assay was used to detect clonal rearrangements of T‐cell receptor gamma locus (TRG) and Immunoglobulin (Ig). Briefly, genomic DNA was isolated from previously deparaffinized and rehydrated 3‐μm‐thick sections using the GIAsymphony DSP DNA Mini Kit (Qiagen, Milan, Italy) following the manufacturer's instructions. PCR was carried out in a final reaction volume of 50 μL, in duplicate, containing 25 μL of 2× HotStarTaq Master Mix (Qiagen), 0.3 μM of each primer, and 5 μL of DNA template. The reaction volume was adjusted to 50 μL with DNase/RNase‐free water. PCR reactions were conducted using the primers set and thermal conditions described by Mochizuki et al and Moore et al,^12^, ^13^ to detect feline clonal Ig and TCR rearrangements, respectively. The PCR products underwent size separation through capillary gel electrophoresis using the QIAxcel Advanced System (Qiagen), employing the QIAxcel DNA High Resolution Kit (Qiagen). The results were represented as electropherograms by the QIAxcel ScreenGel Software 1.5 (Qiagen). For each PARR primer set, results were categorized as clonal (1 or 2 tall clear peaks with minimal polyclonal amplification), polyclonal (fragments distributed across the size range of the primer set, either in a Gaussian distribution or a slightly more skewed distribution), monoclonal within a predominantly polyclonal background (1 or more clear peaks visible within a polyclonal distribution), and negative (no amplification products in the size range).

### 2.5 Statistical analyses

All data were input in an electronic datasheet, and descriptive statistics were calculated. Categorical variables were presented in terms of frequency, while age was expressed as median and range after evaluating data distribution using the Shapiro‐Wilk test.

A binomial logistic regression analysis was conducted to identify potential factors influencing the probability of LN infiltration. The following variables were considered: breed (domestic short hair [DSH], other), sex (spayed female, neutered male), age (years), neoplastic cell size (small, large), immunophenotype (B, T), infiltration pattern (mucosal, transmural), lymphoma subtype (TSTCL, MSTCL, TLBCL, TLTCL, MLBCL).

Breed, sex, age, presence of LN involvement (yes, no), cell size and phenotype were evaluated to determine their predictive potential for intestinal architecture. Additionally, breed, sex, age and presence of LN involvement were evaluated for their predictive capacity for specific lymphoma subtype.

Variables reaching significance in the univariate analysis were included in a multivariate analysis using backward elimination.

All statistical analyses were performed using SPSS v28.0for Windows, with a significance level set at *P* ≤ .050 for all tests.

## 3 RESULTS

Out of 589 histologic reports collected over 6 years, 102 met the inclusion criteria. Eight different breeds were represented, including 78 (77.2%; 95% CI: 68.1%‐84.4%) DSH, 7 (6.9%; 95% CI: 3.2%‐13.8%) Abyssinian, 7 (6.9%; 95% CI: 3.2%‐13.8%) Maine Coon, 3 (3.0%; 95% CI: 0.6%‐8.7%) Bengal, 2 (2.0%; 95% CI: 0.1%‐7.4%) Devon Rex, 2 (2.0%; 95% CI: 0.1%‐7.4%) Norwegian Forest, and 1 (1.0; 95% CI: 0.0%‐5.9%%) each of Persian and Sphinx. Breed was not reported in 1 case. All cats were neutered, including 55 (53.9%; 95% CI: 44.3%‐63.3%) spayed females and 47 (46.1%; 95% CI: 36.7%‐55.7%) castrated males. Median age at diagnosis was 10 years (range, 2‐18 years). The full vaccination history, as well as FIV and FELV status for individual cats, were not available.

Concerning intestinal histopathology, 40 (39.2%; 95% CI: 30.3%‐48.9%) biopsies were classified as TSTCL, 32 (31.4%; 95% CI: 23.2%‐40.9%) as MSTCL, 16 (15.7%; 95% CI: 9.8%‐24.1%) as TLBCL, 8 (7.8%; 95% CI: 3.8%‐14.9%) as TLTCL and 6 (5.9%; 95% CI: 2.5%‐12.5%) as MLBCL. No cases of MLTCL, MSBCL and TSBCL were diagnosed.

Nodal infiltration by lymphoma was found in 44 (43.1%; 95% CI: 33.9%‐52.8%) cases, including 16 (100.0%; 95% CI: 77.3%‐100.0%) TLBCL, 15 (37.5%; 95% CI: 24.2%‐53.0%) TSTCL, 6 (75.0%; 95% CI: 40.1%‐93.7%) TLTCL, 6 (18.8%; 95% CI: 8.5%‐35.7%) MSTCL and 1 (16.7%; 95% CI: 1.1%‐58.2%) MLBCL. Neoplastic cell size and phenotype in the LN consistently matched those found in the corresponding intestinal biopsy.

**FIGURE 1:**
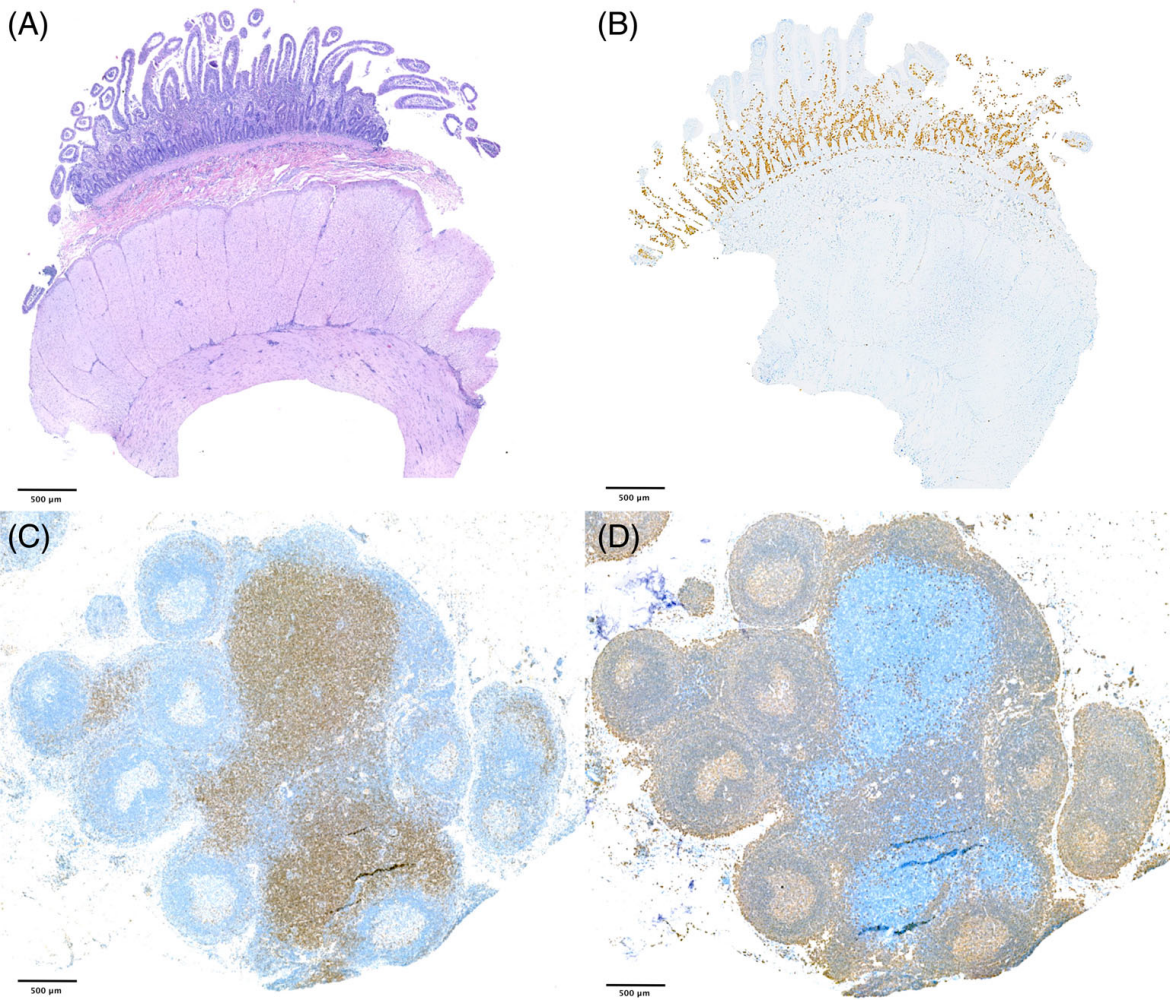
Full‐thickness duodenal biopsy (A, B) and corresponding mesenteric lymph node (LN; C, D) specimen from a cat diagnosed with mucosal small T‐cell lymphoma (MSTCL) without LN infiltration. 1× magnification. (A) Hematoxylin and eosin (HE) staining showing a monomorphic group of small lymphocytes diffusely invading the mucosa. (B) Anti‐CD3 antibody staining showing diffuse and severe infiltration of neoplastic cells within the mucosa. (C) LN immune stained with anti‐CD3 antibody showing mild hyperplastic paracortex. (D) LN immune stained with anti‐CD20 antibody showing moderate increase in follicle size.

**FIGURE 2:**
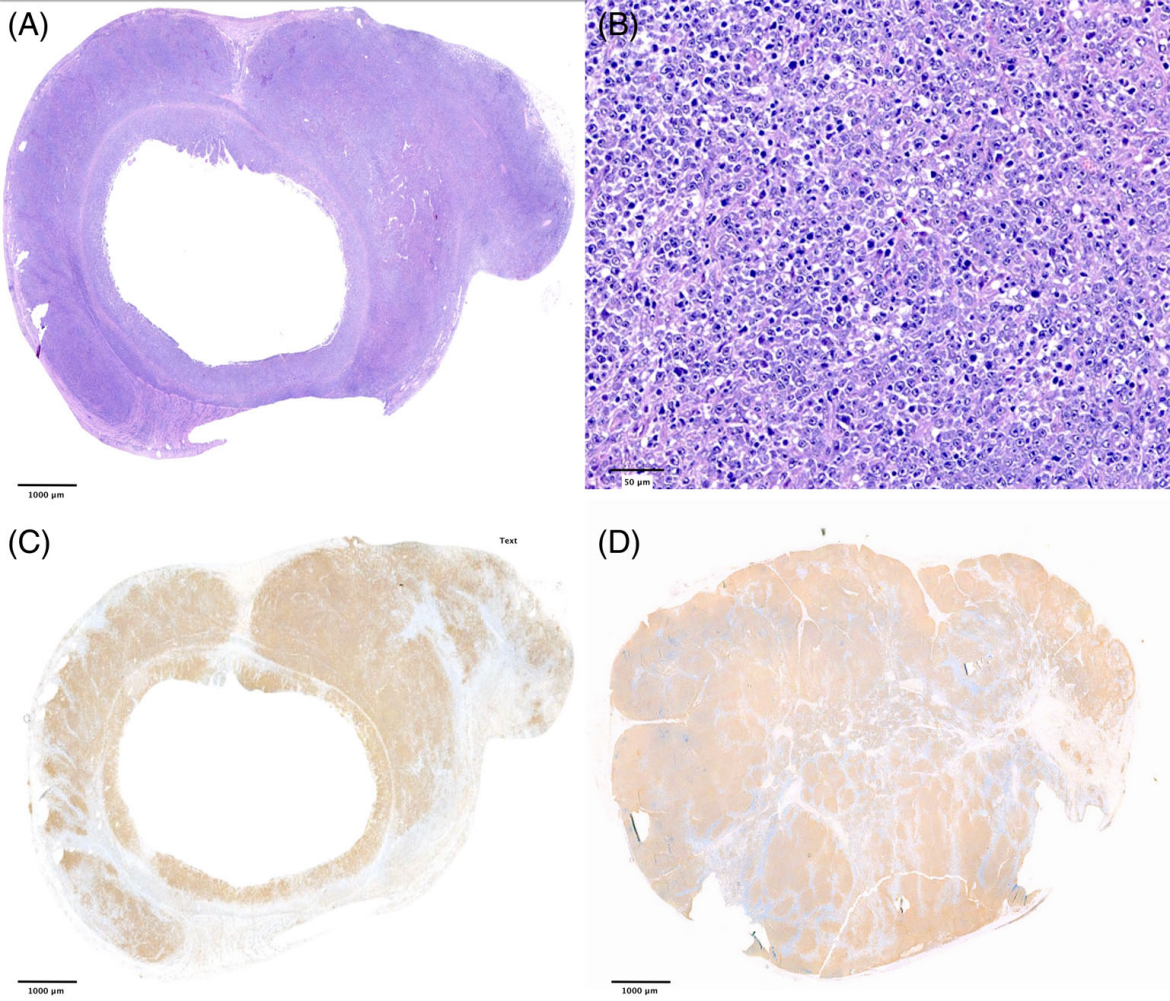
Full‐thickness duodenal biopsy (A‐C) and corresponding lymph node (LN; D) specimen from a cat diagnosed with transmural large B‐cell lymphoma (TLBCL) with LN infiltration. (A) Hematoxylin and eosin (HE) staining showing monomorphic large lymphocytes diffusely invading the mucosa, submucosa, and muscular layer. 1× magnification. (B) HE staining revealing the neoplastic infiltrate consisting of large lymphocytes with pleomorphic nuclear morphology. 40× magnification. (C) Anti‐CD20 antibody staining showing diffuse and severe infiltration of neoplastic cells within the mucosa, submucosa, and muscular layer. 1× magnification. (D) Anti‐CD20 antibody staining showing diffuse and severe infiltration of neoplastic cells of the corresponding mesenteric LN. 1× magnification.

The presence of LN involvement was significantly higher in DSH cats and those with B‐cell phenotype, large cell size or transmural infiltration pattern (Table 1, Figures 1 and 2). Conversely, sex, age and specific lymphoma subtype did not influence the likelihood of LN infiltration (*P* > .050). Only cell size and infiltration pattern retained significance (*P* < .001 and *P* = .002, respectively) in multivariate analysis. Specifically, cats with both independent predisposing factors (large cell size and transmural infiltration pattern) had a 28‐fold higher likelihood of LN infiltration compared to all other cats (odds ratio, 28.00; 95% CI, 6.07‐129.21), while cats lacking both factors had a 28‐fold lower likelihood than all other cats (odds ratio, 0.04; 95% CI, 0.01‐0.17).

**TABLE 1:** Variables significantly affecting the likelihood of having nodal infiltration in 102 cats with intestinal lymphoma.

Variable	Univariate analysis			Multivariate analysis		
	*P*‐value	Odds ratio	95% confidence interval	*P*‐value	Odds ratio	95% confidence interval
Breed	.026			NS		
DSH		3.42	1.16‐10.13			
Other		Ref	Ref			
Phenotype	<.001			NS		
B		6.67	2.22‐20.04			
T		Ref	Ref			
Cell size	<.001			<.001		
Large		7.98	2.97‐21.41		6.83	2.41‐19.40
Small		Ref	Ref		Ref	Ref
Intestinal pattern	<.001			.002		
Transmural		6.07	2.32‐15.82		5.12	1.83‐14.35
Mucosal		Ref	Ref		Ref	Ref

Abbreviations: DSH, domestic short hair; NS, not significant.

The likelihood of transmural intestinal architecture decreased with age but increased with large cell size and the presence of LN infiltration. However, cell size lost significance in multivariate analysis (Table 2). Ultimately, age emerged as the sole significant predictor of the specific lymphoma subtype (*P* = .003), indicating a higher prevalence of MSTCL in younger cats (6 out of 11 cats ≤3 years old).

**TABLE 2:** Variables significantly influencing the probability of transmural lymphoma subtype in 102 cats with intestinal lymphoma.

Variable	Univariate analysis			Multivariate analysis		
	*P*‐value	Odds ratio	95% confidence interval	*P*‐value	Odds ratio	95% confidence interval
Age	.001	0.82	0.73‐0.93	.003	0.82	0.73‐0.93
Cell size	.024			NS		
Large		3.20	1.17‐8.77			
Small		Ref	Ref			
Nodal infiltration	<.001			<.001		
Yes		6.07	2.33‐15.83		6.03	2.20‐16.55
No		Ref	Ref			

Abbreviation: NS, not significant.

A clonality test was performed on 30 LNs diagnosed with reactive LN. Results revealed polyclonality in 19 (63.3%; 95% CI: 45.5%‐78.2%) samples (17 MSTCL and 2 MLBCL), indicating the presence of multiple lymphocyte populations and confirming the histologic diagnosis. Notably, 5 (20.0%; 95% CI: 8.4%‐39.6%) cases of MSTCL displayed monoclonality within a predominantly polyclonal background, suggesting clonal expansion of a lymphocyte subpopulation within a reactive environment. Additionally, 6 (20.0%; 95% CI: 9.1%‐37.7%) cases displayed monoclonal amplification. However, in 3 cats with MLBCL, PARR results contradicted the morphologic diagnosis and the expected lymphoma phenotype; instead, TCR monoclonality was identified. Supplementary Table 2 provides comprehensive details of the PARR results.

## 4 DISCUSSION

This study aimed to investigate whether evaluating the regional LN, rather than the primary intestinal disease, could serve as a diagnostic surrogate for the primary pathology. To this aim, a thorough examination of mesenteric LNs was conducted in 102 cats diagnosed with various intestinal lymphoma histotypes, using histologic and IHC analysis. As hypothesized, the LN, when involved, can assist in diagnosing large cell lymphoma, but not small cell lymphoma.

The current series included 64 cases with transmural lesions (including 24 with large cell morphology) and 38 cases with mucosal lesions (including 6 with large cell morphology). In comparison to findings reported by Moore et al,^2^ our series showed a higher prevalence of cases classified as TSTCL. This difference could be attributed to the method of sampling, with full‐thickness biopsy performed in all cases in our series compared to 70% in the previous study.^2^ The utilization of full‐thickness biopsy allows for a more comprehensive assessment of potential transmural lymphoma infiltration, as opposed to superficial layers examined in endoscopic biopsy. Overall, T‐cell lymphomas were more frequent than those with a B‐cell phenotype (78.4% vs 21.6%, respectively), likely influenced by the study's inclusion criterion focusing on lymphomas primarily affecting the small intestine.

Also, within the B‐cell phenotype category, there were no diagnoses of small‐cell mucosal or transmural lymphomas. This finding is not unexpected, given that the intestinal mucosa comprises the largest lymphoid population in the body, predominantly consisting of T lymphocytes.^19^, ^20^ Finally, large B‐cell lymphomas were more frequently observed as transmural rather than mucosal, in line with previously reported data.^2^

All intestinal biopsies were accompanied by excision of the associated regional LN. As this was a selection from the archive, complete clinical information and imaging findings were not consistently available, making it challenging to determine if the LNs were removed because of macroscopic alterations. Among the 102 examined LNs, 58 (56.9%; 95% CI: 47.2%‐66.1%) showed reactive changes and 44 (43.1%; 95% CI: 33.9%‐52.8%) were infiltrated by neoplastic cells. Large cell lymphomas with nodal involvement accounted for 23 out of 30 cases (76.7%; 95% CI: 58.8%‐88.5%), while small cell lymphomas for 21 out of 72 cases (29.2%; 95% CI: 19.9%‐40.6%). These findings suggest that large cell lymphoma more frequently involve regional LNs more frequently compared to small cell lymphoma. Therefore, excising the LN during surgical biopsy instead of intestinal biopsy, would not yield a definitive diagnosis in the vast majority of small cell lymphoma cases and should therefore be avoided. Conversely, in the case of large cell lymphoma, most LNs were infiltrated by neoplastic cells, thereby facilitating the diagnosis.

In this study, we also aimed to assess whether PARR, in cases of reactive LN, could aid in identifying neoplastic cells, assisting in the diagnosis of intestinal lymphoma in cats. PARR accurately identified 63% of reactive LNs, but it is concerning that, in the remaining cases, it detected a clonal population suggestive of cancer. It is plausible that a predominant monoclonal population was overlooked during histologic examination and subsequent IHC. However, it is crucial to emphasize that our study exclusively included reactive LNs characterized by discrete follicles with enlarged germinal centers. These follicles were composed of lymphoblasts and immature lymphocytes surrounded by a mantle of mature lymphocytes. Additionally, other observed lesions included the presence of a mixture of histiocytes, plasma cells, and immunoblasts, expanding paracortical regions without effacing follicles.^17^ The discordance between genetic characteristics and morphology poses a diagnostic challenge, highlighting the complexity of such cases and emphasizing the need for expert interpretation within the broader clinical context. However, achieving accurate results in interpreting PCR findings faces challenges.

In 3 cats diagnosed with large B‐cell lymphoma confined to the mucosa, inconsistencies between histologic and immunohistochemical analysis and PARR results were identified in the LNs. The absence of large CD20+ tumor cells indicates the unlikely infiltration of the LNs by the tumor in these instances. Conversely, the presence of monoclonality in TCR could be influenced by a specific subset of T‐cell lymphocytes stimulated by the tumor microenvironment, leading to alterations in clonal composition. Nevertheless, further investigations are necessary to analyze the diversity and arrangement of TCR clones within the intestinal lymphoma microenvironment in cats to validate this hypothesis.^21^ Overall, the findings from PARR once again emphasize the notion that relying solely on clonality might not provide adequate insight into the phenotype of the lymphoid population.^9^

Intriguingly, our study revealed a higher prevalence of MSTCL diagnoses in younger cats. Typically, cats with MSTCL are older, and the pathogenesis is linked to prolonged inflammatory stimulation.^2^, ^13^ This unexpected finding suggests the need to explore alternative factors driving oncogenesis, including abnormalities in DNA methylation or germ line mutations.^22^ It also raises questions about the potential role of germline pathogenic variants that might predispose these cats to lymphoma. While genomic characterization has become a valuable tool for guiding cancer screening and diagnosis in humans, and is increasingly utilized in dogs, studies in cats are currently lacking.^23^, ^24^ Investigating the variations in specific DNA sequences variations between tumor samples and reference genomes or matched normal DNA could importantly advance our understanding of this intricate disease in cats.^25^

There are a few limitations that need to be acknowledged in this study. First, it was retrospective in nature, relying on archival tissues without access to clinical, imaging, therapeutic, or follow‐up data. While mucosal small T‐cell lymphomas have been associated with a favorable prognosis when treated with chlorambucil and prednisone, the literature lacks information regarding the transmural form of small T‐cell lymphomas.^4^, ^5^ It is plausible that some cases reported in prior studies might have had transmural involvement, albeit not confirmed because of sampling limitations during endoscopy. In future research, it will be necessary to determine whether transmural small T‐cell lymphomas share a similar prognosis to their mucosal counterpart or exhibit a more aggressive biological behavior.

Second, despite our inclusion criteria targeting clear‐cut mucosal lymphomas with a minimum of 5 plaques in each biopsy, there remains a possibility of some cases being misclassified as inflammatory bowel disease. This uncertainty also extends to LN biopsies, where despite histological and immunohistochemical consistency with lymphoma or reactive pattern, the presence of early‐stage small‐cell lymphomas cannot be entirely ruled out. Unfortunately, PARR clonality testing, as reported previously, is not always definitive in distinguishing between cancer and a reactive condition.^1^, ^3^ Both false‐positive and false‐negative cases have been documented in the literature,^1^, ^3^ and our study similarly encountered ambiguous findings, diagnosing 11 out of 30 cases interpreted as reactive based on histology and IHC as lymphoma. As suggested by Marsilio et al,^26^ it is important to consider the possibility of lymphocyte trafficking phenomenon within the lymph nodes, which could contribute to positive PARR results. In this scenario, the migration of a small number of neoplastic lymphocytes might result in the detection of clonal TRG rearrangements even in the absence of relevant histopathologic alterations.

In conclusion, our study reaffirms the prevalence of T‐cell lymphomas, especially transmural cases, in the small intestine of cats when strict inclusion criteria are applied. It underscores the critical role of full‐thickness biopsies in identifying transmural infiltration and highlights the risk of misdiagnosing small cell or mucosal lymphomas when relying solely on mesenteric LN sampling for histologic, IHC and clonality analysis. The prognostic implications of neoplastic infiltration in LNs remain uncertain, posing a question for future studies.

## CONFLICT OF INTEREST DECLARATION

Authors declare no conflict of interest.

## OFF‐LABEL ANTIMICROBIAL DECLARATION

Authors declare no off‐label use of antimicrobials.

## INSTITUTIONAL ANIMAL CARE AND USE COMMITTEE (IACUC) OR OTHER APPROVAL DECLARATION

Authors declare no IACUC or other approval was needed.

## HUMAN ETHICS APPROVAL DECLARATION

Authors declare human ethics approval was not needed for this study.

## Supporting information


**Supplementary Table 1.** antibody used for immunohistochemical analysis of 102 intestinal biopsies and respective mesenteric lymph nodes from cats with intestinal lymphoma.

**Supplementary Table 2.** Histopathological results of intestinal biopsy and matched mesenteric lymph nodes in 102 cats with intestinal lymphoma.
