# Supplementary material for: The diagnostic relevance of mesenteric lymph node biopsy in small intestinal lymphoma in cats
Source: J Vet Intern Med. 2024 Jun 10;38(4):2316–23. doi: 10.1111/jvim.17095 (PMC11256130; doi:10.1111/jvim.17095)
Supplement: Supplementary file 1 — Supplementary Table 1. antibody used for immunohistochemical analysis of 102 intestinal biopsies and respective mesenteric lymph nodes from cats with intestinal lymphoma. [file JVIM-38-2316-s002.docx]

**Supplementary Table 1:** antibody used for immunohistochemical analysis of 102 intestinal biopsies and respective mesenteric lymph nodes from cats with intestinal lymphoma

| **target** | **specificity** | **clone** | **source** |
| --- | --- | --- | --- |
| CD3 | T-cells | Monoclonal mouse anti-human  F7.2.38 | Dako, Glostrup, Denmark |
| CD20 | B-cells | Monoclonal rabbit anti-human  SP32 | Abcam, Cambridge, UK |
